# Evaluating Urban Building Damage of 2023 Kahramanmaras, Turkey Earthquake Sequence Using SAR Change Detection

**DOI:** 10.3390/s23146342

**Published:** 2023-07-12

**Authors:** Xiuhua Wang, Guangcai Feng, Lijia He, Qi An, Zhiqiang Xiong, Hao Lu, Wenxin Wang, Ning Li, Yinggang Zhao, Yuedong Wang, Yuexin Wang

**Affiliations:** School of Geosciences and Info-Physics, Central South University, Changsha 410083, China; csuxhgd@csu.edu.cn (X.W.); lijiahe@csu.edu.cn (L.H.); anqi27@csu.edu.cn (Q.A.); zqxiong_flhs@csu.edu.cn (Z.X.); lu_hao@csu.edu.cn (H.L.); wangwx@csu.edu.cn (W.W.); 225012160@csu.edu.cn (N.L.); zhaoyg@csu.edu.cn (Y.Z.); ydwang@csu.edu.cn (Y.W.); wangyuexin@csu.edu.cn (Y.W.)

**Keywords:** SAR, change detection, coherence, building damage evaluation, earthquake

## Abstract

On February 6, 2023 (local time), two earthquakes (Mw7.8 and Mw7.7) struck central and southern Turkey, causing extensive damage to several cities and claiming a toll of 40,000 lives. In this study, we propose a method for seismic building damage assessment and analysis by combining SAR amplitude and phase coherence change detection. We determined building damage in five severely impacted urban areas and calculated the damage ratio by measuring the urban area and the damaged area. The largest damage ratio of 18.93% is observed in Nurdagi, and the smallest ratio of 7.59% is found in Islahiye. We verified the results by comparing them with high-resolution optical images and AI recognition results from the Microsoft team. We also used pixel offset tracking (POT) technology and D-InSAR technology to obtain surface deformation using Sentinel-1A images and analyzed the relationship between surface deformation and post-earthquake urban building damage. The results show that Nurdagi has the largest urban average surface deformation of 0.48 m and Antakya has the smallest deformation of 0.09 m. We found that buildings in the areas with steeper slopes or closer to earthquake faults have higher risk of collapse. We also discussed the influence of SAR image parameters on building change recognition. Image resolution and observation geometry have a great influence on the change detection results, and the resolution can be improved by various means to raise the recognition accuracy. Our research findings can guide earthquake disaster assessment and analysis and identify influential factors of earthquake damage.

## 1. Introduction

On 6 February 2023, strong earthquakes occurred in Kahramanmaras Province and Gaziantep Province in south-central Turkey, including two ≥Mw7.0 earthquakes. According to GCMT data, the first earthquake occurred at 4:17 a.m. local time, with a magnitude of Mw7.8 and an epicenter located in Kahramanmaras Province (37.47° E, 37.56° N) at a depth of 15 km. The second earthquake occurred at 1:24 p.m. local time with a magnitude of Mw7.7 and an epicenter located in the Gaziantep Province (37.22° E, 38.11° N) at a depth of 12 km. These two earthquakes are larger than Mw7.0, and their epicenters are about 96 km apart. They are the “double shock earthquake” in the swarm earthquake. Most buildings in southern Turkish cities have unreinforced brick masonry structures and low-rise concrete frames, so thousands of buildings collapsed in the earthquake, causing extensive casualties and damages [1,2]. The identification and statistical analysis of damaged buildings are crucial for disaster relief, assessment and post-disaster reconstruction.

For the 2023 Turkey earthquake sequence, some teams used high-resolution optical images to extract damaged buildings in the earthquake area. The Microsoft team partnered with Turkey’s Ministry of Interior Disaster and Emergency Management Presidency (AFAD) and used high-resolution optical images and artificial intelligence recognition technology to identify and evaluate the extent of building damage in four severely affected cities and obtained the damage ratio of urban buildings [3]. Huang Xin’s team used high-resolution Maxar satellite images (resolution of 0.5 m) and semantic segmentation network to locate and count collapsed buildings in six earthquake-affected cities [4]. These two teams both applied artificial intelligence and deep learning technology to get accurate and reliable change information of buildings from high-resolution optical images, which are difficult to be widely applied and are seriously limited by weather conditions. Sentinel-1 data are free, medium-resolution SAR images distributed by ESA. They can be used in large-scale applications because of their all-day and all-weather earth observation and easy accessibility. The SAR image contains rich amplitude information and complex information, supporting both change detection and surface deformation analysis.

To extract the information of earthquake-damaged buildings, some scholars determined the extent of building damage by investigating the relationship between backscattering coefficient, coherence and intensity correlation [5,6]. In SAR images, change detection methods are mainly based on amplitude and phase coherence. By identifying the change of texture features of amplitude images, filtering out noise and constructing difference image, scholars get the change of buildings [7,8,9,10,11]. Coherence network construction and interference processing have been used to obtain coherence change images and extracted buildings [12,13,14,15]. Machine learning and deep learning also have been applied to the field of building change detection [16,17,18]. However, how to effectively obtain training samples, learn effective features and reduce false changes require further study [19,20].

In this study, we use the proposed change detection method and the descending and ascending Sentinel-1 data to identify the urban building damage caused by the 2023 Turkey earthquake sequence. Utilizing the surface deformation results and building damage information, we also analyzed the seismic geological factors on buildings. Additionally, we discussed the feasibility of using medium-resolution SAR images to identify damaged buildings in the earthquake environment. This cost-effective method is simple, responsive and requires minimal image usage. It could be an essential technology for identifying large-area damage of buildings by large earthquake.

## 2. Method

SAR change detection is a powerful means of earthquake damage assessment. Buildings are stable objects with high scattering intensity, producing stable echo signals in SAR images. If a building is destroyed, its geometric shape changes, so does its echo signal. By analyzing the difference of images’ intensity before and after the earthquake, the damaged region can be identified, providing information for disaster relief.

### 2.1. Amplitude Difference Extracts Damaged Buildings in the Affected Urban Area

There are a variety of SAR image change detection methods. The most commonly used are the difference method, the ratio method [21], the logarithmic ratio method [22,23,24], etc. These are algebraic methods, which directly convert the amplitude information into the backscattering coefficient and use the images before and after the change to construct the difference image to extract the change. The methods are simple and have wide application. The difference method uses the difference of one point on two registered images to obtain the difference image. The absolute value of the difference is proportional to the degree of change. The backscattering coefficient of the damaged buildings changes greatly. Using the difference method to construct the difference image can highlight the damaged buildings and help to extract the change area. SAR amplitude images have obvious salt and pepper noises that affect the accuracy of the difference method. It is necessary to filter and denoise the image before applying the difference method.

### 2.2. Coherence Change Detection Extracts Damaged Buildings in the Affected Urban Area

**Figure 1:**
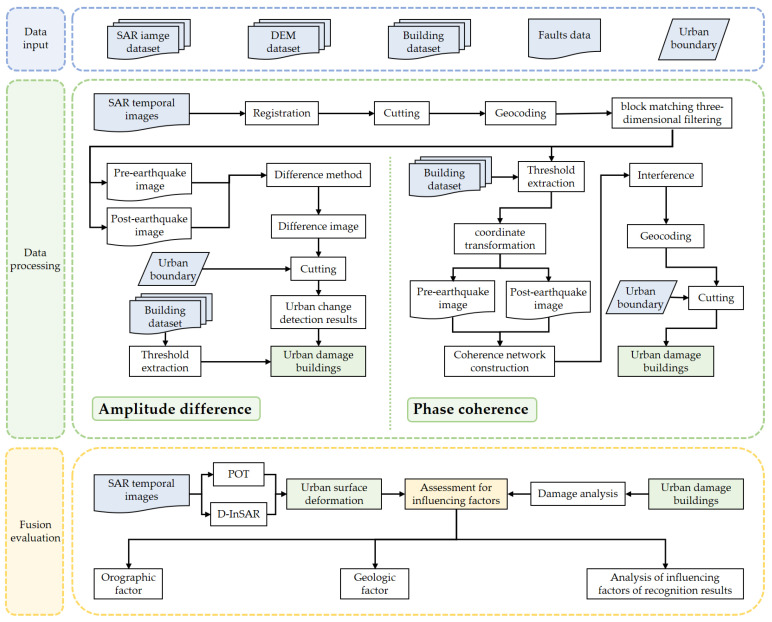
Flowchart used in this study.

Change detection based on SAR image coherence is an effective method to extract building information, using the complex information of SAR images. SAR image coherence measures the similarity between two SAR signals, to characterize the change extent of ground objects. It is often used as an accuracy index of interferometric phase. When the phase signals of two SAR images are similar, they are considered to be coherent. In SAR images, stable objects such as rocks and buildings often show high coherence, while vegetation, water bodies and unstable objects show low coherence [11]. For the seismic damage area, the damaged buildings show low coherence, so the damaged buildings can be identified by coherence change detection in the building-intensive area (Figure 1).

## 3. Data Processing and Results

### 3.1. Dataset

**Figure 2:**
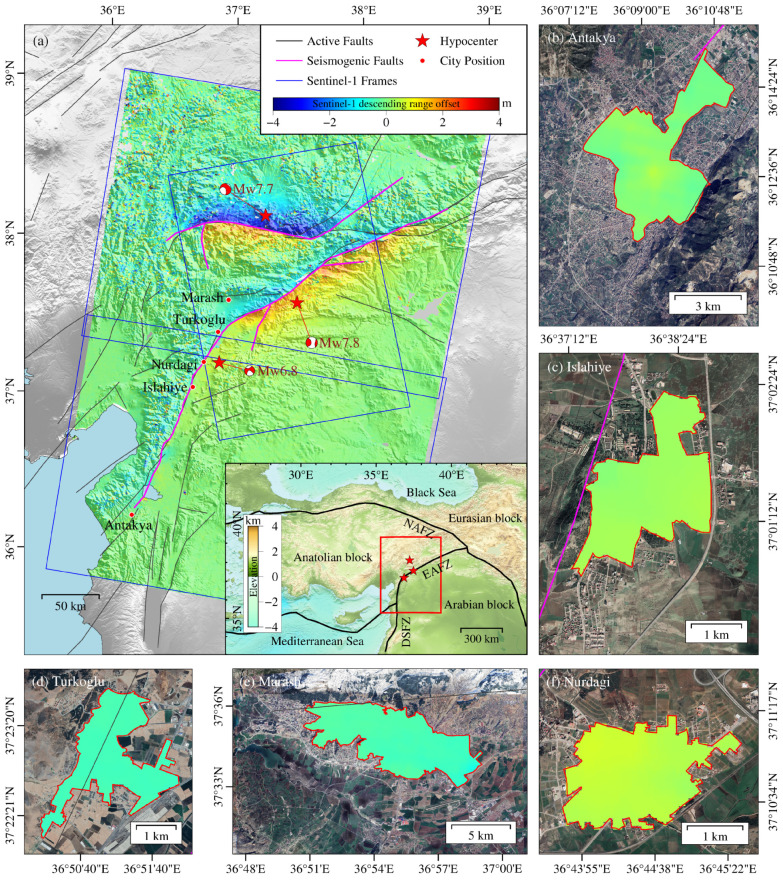
Surface deformation of the five urban areas caused by the 2023 Mw7.7 and Mw7.8 Turkey earthquakes obtained by POT. (**a**) Surface deformation of central southern Turkey. The blue box indicates the Sentinel-1 image coverage; the dark gray lines are active faults; the magenta lines are seismogenic faults; and the red pentagrams indicate the epicenter of the two main earthquakes and the Mw6.8 aftershock. The epicenter and focal mechanism are cited from GCMT. The red dots are the location of the affected cities. The inset shows the regional seismotectonic background: The red box is the study area shown in (**a**); the black line is the fault zone; the north one is North Anatolian fault zone (NAFZ); and the south are East Anatolian fault zone (EAFZ) and the Death Sea fault zone (DSFZ). The red pentagonal stars are the epicenters, (**b**–**f**) zoom-in of the deformation map of the five cities. The red polygon delineates the urban boundary.

We selected six Sentinel-1 Single Look Complex (SLC) images for the study, including two ascending images and four descending images located at Track21, two in Frame 465 and two in Frame 471. The revisited cycle of a single satellite is 12 days. The revisited cycle and wide coverage allow large-scale surface deformation monitoring. The images cover central and southern Turkey, and the image time spans from 29 January 2023 to 28 February 2023, covering the time that the earthquake happened (Figure 2 and Table 1). To keep the original resolution, images are processed by 1:1 (range:azimuth) multi-look operation. The post-earthquake images are obtained four days after the earthquake, so they may contain few affects caused by other factors, such as afterslip. Combining the fault distribution and earthquake disaster situation, we selected five severely affected cities in central and southern Turkey as the study area, namely Islahiye, Antakya, Turkoglu, Nurdagi and Marash. Optical images show that the buildings in these five cities were damaged or even collapsed. Due to the lack of official urban boundary data, this experiment takes the relatively dense area of buildings as the urban area by manual delineation.

As Figure 2 shows, the five cities are all located near active faults. Islahiye and Nurdagi are located at the seismogenic fault; Turkoglu is close to the seismogenic fault; and Antakya is at the south end of the seismogenic fault.

### 3.2. Data Procecssing

Before using change detection (the difference method) to identify damaged buildings in urban areas, the data is first preprocessed. Firstly, the registered images are cropped according to the city scope to make each slice certain one city. Then, the SAR coordinate system is converted to the geographic coordinate system. Finally, we use the block matching three-dimensional filtering (BM3D) to remove the speckle noise. The difference image is obtained by subtracting the preprocessed pre-earthquake and post-earthquake images. Since buildings are concentrated in an urban area, the artificial urban coverage is used to cut them to focus on the damage of urban buildings. The results contain buildings and other features. We used the existing building data set to calculate the backscattering coefficient of buildings so as to determine the threshold, which is finally used to detect the building changes. Then, the damage of urban buildings is obtained.

When the image coherence is used to identify damaged buildings in urban areas, we first use thresholds to extract buildings in the image and convert the image into the SAR coordinate system. Then, we used GAMMA software (version 2020) to construct the coherence network of the extracted building images. Then, we obtained the coherence results through interference processing. After converting the coherence results from the SAR coordinate system to the geographic coordinate system, we also cropped them to obtain the urban coherence results. Because stable buildings show high coherence and damaged buildings show low coherence, the damaged buildings can be extracted by threshold.

We also used POT and D-InSAR technique to obtain the surface deformation. POT is not affected by phase decoherence noise, so it has a great advantage in obtaining large-magnitude surface deformation [25,26,27,28]. For urban areas with small surface deformation, D-InSAR technology is used to obtain surface deformation [29]. The obtained deformation offset has various errors. Then, we employed the first-order polynomial to remove the trend error and the mean elimination method to remove the stripe error [30]. We extracted the surface deformation results according to the artificial urban coverage and obtained the urban surface deformation of each city. The detailed process parameters are shown in Table 2.

For urban areas with small deformation, D-InSAR technology was used to obtain surface deformation. Firstly, we used GAMMA software to perform registration and differential interference processing on the image pair and obtained the interferogram. The multi-look number of interferogram pair is 5:1 (range: azimuth).

### 3.3. Results

**Figure 3:**
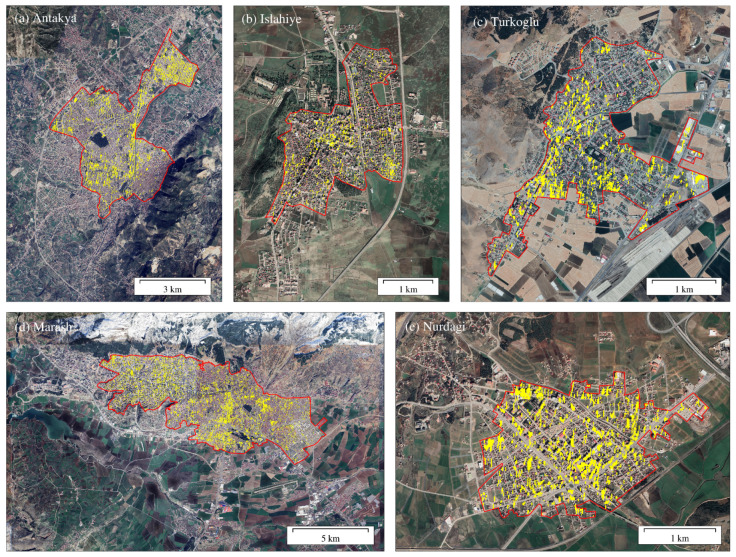
Urban building damage identification results with descending data. The red polygon delineates the urban boundary. Yellow-filled polygons are the identified damaged buildings.

The regions with damaged buildings are scattered throughout the urban area. Since the urban area is mainly composed of buildings, the ratio of the damaged building area to the urban area is used as the damage ratio. Nurdagi, Marash, Antakya, Turkoglu and Islahiye ranked from the highest to the lowest in terms of damage ratio. As shown in Figure 3, Nurdagi urban area had the highest proportion of damage in the five cities, 18.93%. In the city, the buildings were mostly damaged, and many single-family buildings were razed to the ground. Marash has the second highest damage ratio of 17.37%. The urban center was the most severely affected, with a large number of houses turned into ruins. As the capital of the Kahramanmaras Province, Marash has a developed economy, transportation and a large residential population. Thus, the earthquake had a great impact on local residents. In Antakya, most damaged buildings are concentrated in the northeast, mainly low self-built houses with a low seismic coefficient. The damaged buildings in the city center are distributed along the main road. Many high-rise buildings collapsed, resulting in a damage ratio of 14.28%. Most of the buildings damaged in Turkoglu are located in the western part of the city, consisting of single, small and medium-sized buildings, with a damage ratio of 12.79%. The building damage in Islahiye is concentrated in the central and southeastern region and had the smallest proportion of damage among the five cities at 7.59% (Table 3).

## 4. Discussion

### 4.1. Comparative Study on the Damaged Building Identification Results

#### 4.1.1. Comparing Our Results with High-Resolution Optical Images

**Figure 4:**
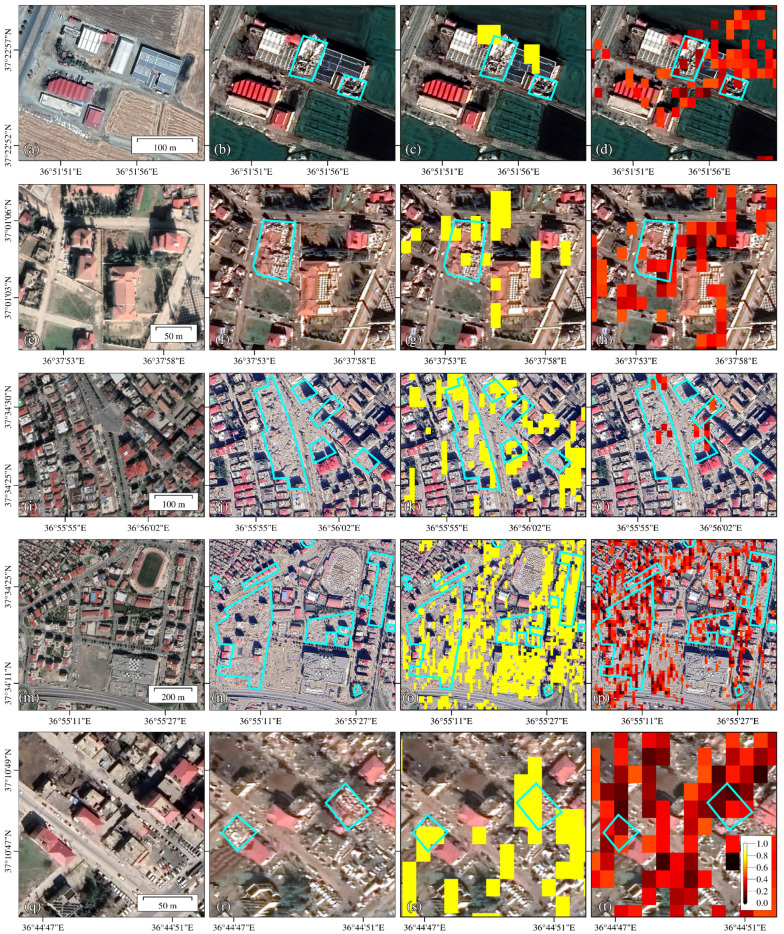
Comparison of the building damage identification results with optical images in urban areas. The first column is the pre-earthquake optical images; the second column is the post-earthquake optical images superimposed damage area contour (cyan polygon). The third column is the post-earthquake optical image superimposed damage area contours and recognition results (yellow filled polygon), and the forth column is the post-earthquake optical image superimposed damage area contours and coherence results. (**a**–**d**) is in Turkoglu; (**e**–**h**) is in Islahiye; (**i**–**p**) is in Marash; and (**q**–**t**) is in Nurdagi.

In this study, the recognition results are verified by the 0.15-m high-resolution optical images of the five cities acquired pre-earthquake and post-earthquake [31]. As shown in Figure 4a,b, the white roof house in the middle of the area collapsed (larger cyan box in Figure 4b), and the southeast corner of the gray roof house collapsed (smaller cyan box in Figure 4b). The change detection recognition results successfully identified the damaged buildings in the area, which are denoted by darker red in Figure 4d. Of course, errors like geocoding, would cause small position deviation between the recognition result and the optical image, but it does not change the location of the damaged building area. Therefore, this study temporarily ignores the errors. In order to extract building changes in the study area as much as possible, the building threshold is set at a low level, resulting in the recognition results containing other changed features. For example, in Figure 4n, change detection recognizes building damages and the changes in the parking lots located in the middle and south of the area, because the parking lots have many motor vehicles, whose shells are strong scatterers. The change of the number and position of these vehicles can cause amplitude changes as large as those of the building changes in the SAR image. Thus, such interference is difficult to remove. Coherence change detection can solve this problem. As shown in Figure 4p, the identified targets in the south and middle are sparser than those in Figure 4o, because coherence change detection eliminates the location changes of sporadic vehicles that show decorrelation. In Figure 4r, damaged buildings are detected on both the west and east sides of the area. These results show that the proposed method is feasible and reliable. If we use high-resolution images (such as 1 m or 3 m), the proposed method can detect the change of single buildings and get high accuracy change detection results.

#### 4.1.2. Comparing Our Results with the Results Obtained by the Microsoft Team

After the earthquake in Turkey, the Microsoft team partnered with Turkey’s Ministry of Interior Disaster and Emergency Management Presidency (AFAD) to use high-resolution optical images (30 cm and 50 cm) and artificial intelligence recognition technology to assess building damage in four disaster-stricken cities, Nurdagi, Marash, Turkoglu and Islahiye. They categorized the buildings as either damaged or undamaged and calculated the damage ratios for each city: 7.30%, 7.44%, 4.85% and 2.48% They also estimated the number of people affected by the disaster according to the population density [3]. These results serve as vital verification data for our study.

**Figure 5:**
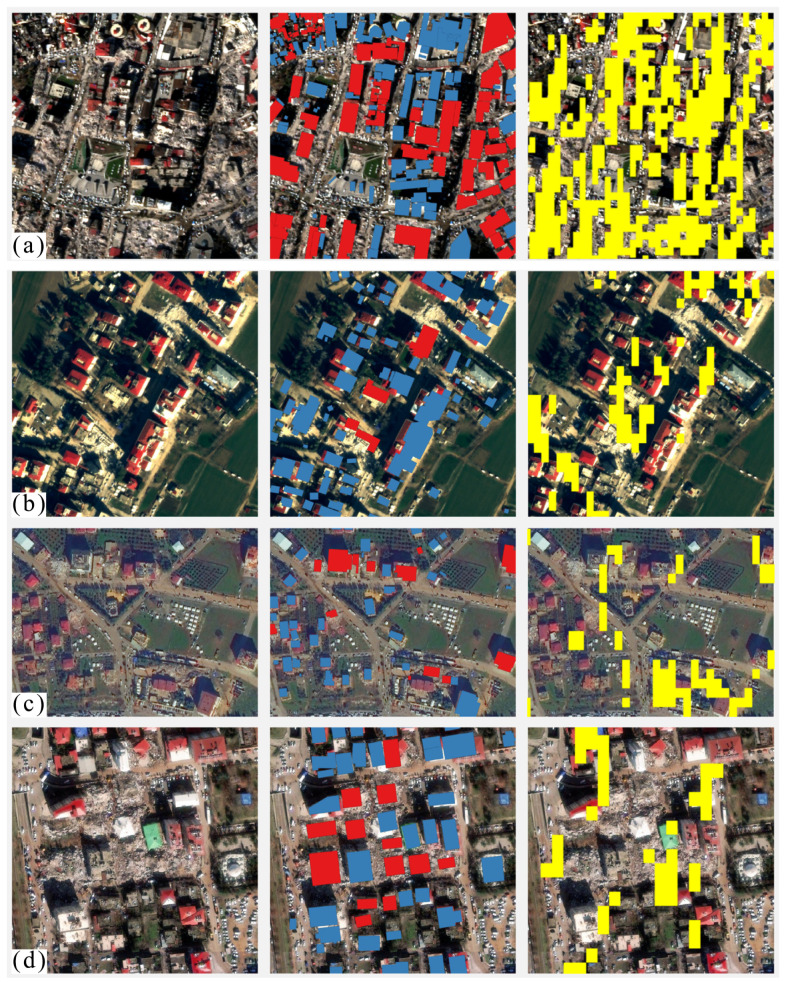
Comparison of urban building damage identification results with Microsoft artificial intelligence identification results. The first column is the post-earthquake optical images; the second column is the post-earthquake optical images superimposed on the artificial intelligence identification results of the Microsoft team (red block is the damaged building; blue block is the undamaged building). The third column is the post-earthquake optical images superimposed on the identification results in this study. (**a**) is in Marash; (**b**) is in Turkoglu; (**c**) is in Nurdagi; (**d**) is in Islahiye.

**Figure 6:**
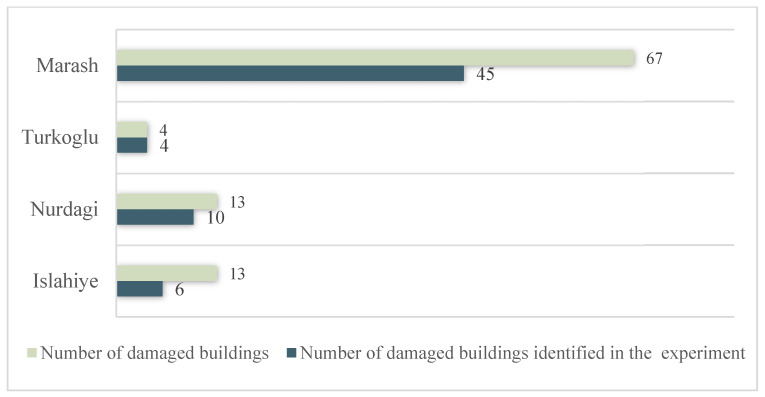
Building damage identification results. The number of damaged buildings comes from the artificial intelligence identification results of the Microsoft team.

In this study, the ranking of the urban building damage ratio is similar to the ranking obtained by the Microsoft team. We compared our results with their published identification results in four local areas. In Marash, we identified 45 out of 67 damaged buildings (Figure 5 and Figure 6). In Turkoglu, our recognition results overlapped with each artificial intelligence recognition result. In Nurdagi, most buildings are small and low-rise, and we detected 10 out of the 13 damaged buildings identified by artificial intelligence. In Islahiye, we identified 6 out of 13 buildings recognized by artificial intelligence. Overall, we detected 65 out of the Microsoft team’s total of 97 damaged buildings, with an accuracy rate of 67%.

Using high-resolution optical images and artificial intelligence technology, the Microsoft team could accurately identify individual buildings in the earthquake area and clearly distinguish damaged buildings. These results are based on high-resolution optical images and robust artificial intelligence recognition algorithms, which are expensive. Additionally, bad weather conditions such as clouds and fog after the earthquake cause difficulties for obtaining effective optical data in time. Thus, this method cannot be widely promoted. The method proposed in this paper aims at identifying large-scale urban damage. Using the free distributed Sentinel-1 images that are not influenced by weather, it can respond quickly after the earthquake and can provide data support for post-disaster rescue and overall disaster assessment. Image resolution plays a decisive role in the recognition results. The method proposed can evaluate the urban building damage as a whole but is not suitable for the extraction of single damaged buildings. Using higher resolution images, this method can obtain higher accuracy detection results, especially for single buildings.

### 4.2. Correlation Analysis of Surface Deformation and Damaged Building Identification Results

For analyzing the influencing factors of building damage, many scholars focus on the buildings themselves, such as structural types and construction period [32,33]. Seismic factors are seldom considered. Here, we conducted a correlation analysis between surface deformation and damaged building identification results to explore the influence of topography, geological structure and other factors on building damage.

**Figure A1:**
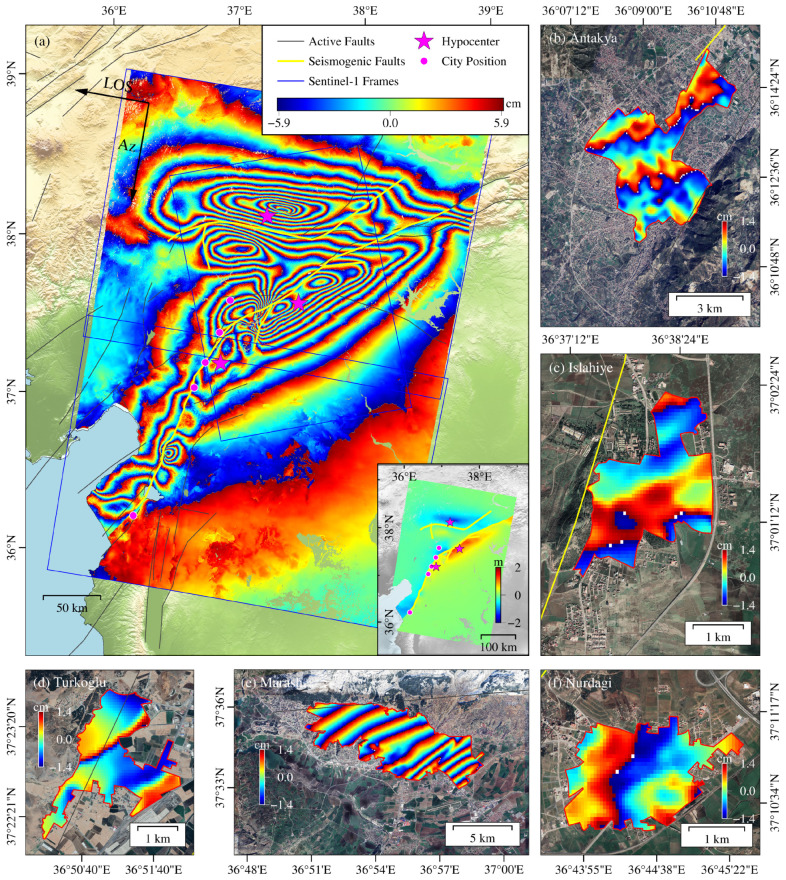
Surface deformation of the five urban areas caused by the 2023 Mw7.7 and Mw7.8 Turkey earthquakes which were obtained by D-InSAR. (**a**) Surface deformation of central southern Turkey. The blue box indicates the Sentinel-1 image cover; the dark gray lines are active faults; the yellow lines are seismogenic faults; and the magenta pentagrams indicate the epicenter of the two main earthquakes and the Mw6.8 aftershock. The epicenter and focal mechanism are cited from GCMT. The magenta circles with white outline are the location of the affected cities. The inset shows surface deformation results with the unwrapping of central southern Turkey: the yellow lines are seismogenic faults, and the magenta pentagrams indicate the epicenter of the two main earthquakes and the Mw6.8 aftershock. The magenta dots are the location of the affected cities. (**b**–**f**) zoom-in of the deformation map of the five cities. The red polygon delineates the urban boundary.

We obtained the surface deformation of the earthquake by both D-InSAR and POT methods. As shown in Figure 2 and Figure A1 in the Appendix A, the surface deformation of this double shock earthquake is featured by left-lateral strike-slip fault movement. The horizontal deformation on both sides of the two seismogenic faults is antisymmetric, and the maximum deformation reaches 5.12 m. The north fault is Cardak fault and Dogansehir fault [34,35,36,37,38]. On the Cardak fault, the deformation is larger on the north side than the south side. On the Dogansehir fault, the deformation is larger on the west side than that on the east side. The south fault is located on the East Anatolian Fault Zone, and the deformation magnitude on the southeast side is larger than that on the northwest side. The large surface deformation magnitude indicates great energy release. The deformation field around the southern fault extends from about 38.5° E, 38° N to 36° E, 36° N, and the cities near the faults are affected by earthquakes. 

Table 4 displays the average surface deformation of the damaged regions in the five selected cities, which is calculated based on building damage identification. We calculated the surface deformation values, and obtained the urban surface deformation of five cities by averaging for subsequent analysis. Turkoglu, Marash, Nurdagi, Islahiye and Antakya ranked highest to lowest in terms of average surface deformation. Turkoglu, located close to the seismogenic fault (the East Anatolian Fault Zone) and epicenter of Mw6.8, had an average surface deformation of 0.50 m. Marash, situated close to the two epicenters of Mw7.7 and Mw7.8 and between the north and south seismogenic faults, had a large average surface deformation of 0.46 m, as it was affected by the two earthquakes. The average surface deformation of the urban area of Nurdagi is 0.46 m, and it is larger in the northwest and smaller in the southeast. As Figure 2a,d shows, Nurdagi is the closest city to the Mw6.8 earthquake, and the seismogenic fault is located on the northwest side of the urban area. The areas closer to the seismogenic fault and epicenter have higher deformation values. In addition, the areas with large surface deformation corresponded with large slip on the fault plane, indicating that segments with greater slip had greater energy releases. Islahiye, also located near the seismogenic fault, had an average surface deformation of 0.28 m. Antakya had the smallest average surface deformation of 0.09 m, as it situated the farthest from the epicenter and is located at the south end of the fault segment.

**Figure 7:**
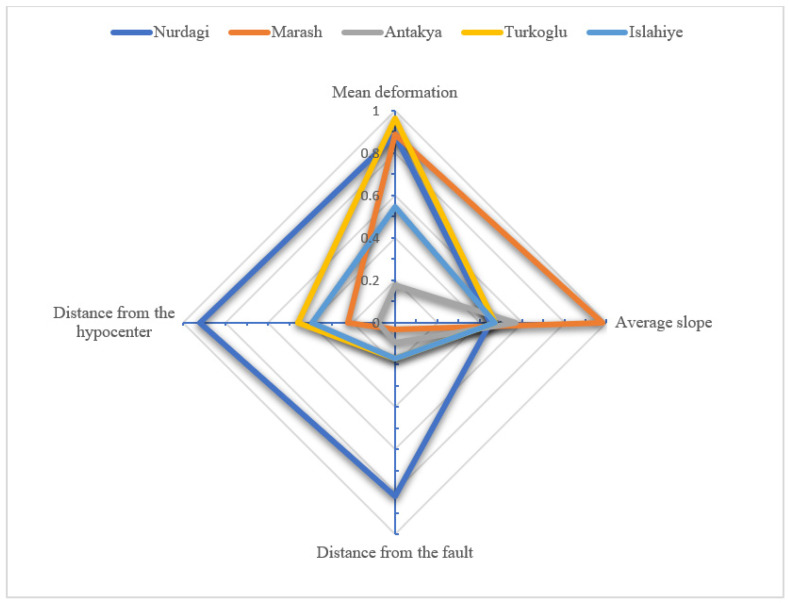
Radar chart of influencing factors of building damage.

We used SRTM 30-m resolution digital elevation data to determine the average slope of the five cities as well as to calculate their distance from both the epicenter and the seismogenic faults. This provided us with the topographic and geological information presented in Table 5 and the radar chart (Figure 7). The distance between the city center and the Mw6.8 earthquake epicenter is selected as the distance between the city and the epicenter as this earthquake is closer to the five cities than the other two earthquakes. The radar chart used four dimensions: magnitude of surface deformation, average slope, distance between the urban center and the seismogenic faults, and distance between the urban center and the epicenter. Each influencing factor is normalized from 0 to 1. The distance between the urban center and the seismogenic fault, and the distance between the urban center and the epicenter are calculated based on reciprocals. Each city is assigned a color. The farther away from the center point of the radar chart in the surface deformation magnitude and the average slope dimension, the greater the surface deformation magnitude and the average slope is. The farther away from the center point of the radar chart in the distance between the urban center and the seismogenic fault and the distance between the urban center and the epicenter, the closer the urban center from the seismogenic fault and the epicenter. The area of the color polygon represents the contribution of the four factors to the urban building damage ratio. The larger the area, the greater the impact.

As Figure 7 shows, Nurdagi has the highest ranking among the five polygons in terms of area, followed by Marash, Turkoglu, Islahiye and Antakya. In addition to Antakya, the polygon area ranking of the remaining cities is the same as the ranking of the urban building damage ratio. These findings suggest that these four factors have impacts on urban buildings damage. The average surface deformation is the largest in Nurdagi, which is the closest to the seismogenic fault and epicenter. But its average slope is small. The Marash urban area has a high average slope and surface deformation but is far from the seismogenic fault. The Turkoglu urban area has a large average surface deformation, is close to the epicenter, but has a small average slope. The impact of the four factors on the damage of Islahiye urban area buildings is relatively average. Although Antakya is the farthest from the seismogenic fault and the epicenter and has the smallest average surface deformation, its buildings were seriously damaged, due to its location at the end of the seismogenic fault and the effects of seismic energy release.

**Figure 8:**
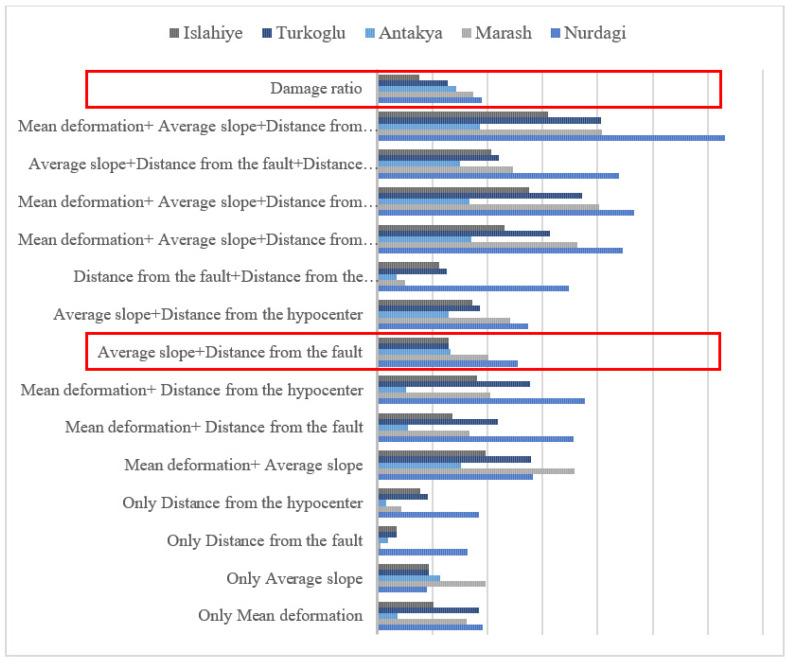
Comprehensive analysis chart of influencing factors. The red box above is the range of the damage ratio of the five cities. The following red box is the range of the superposition results of the average slope and the distance between the urban center and the seismogenic fault of the five cities.

The four factors are superimposed on each other to obtain a comprehensive analysis map of the factors (Figure 8). As shown in the red box, the superposition results of the average slope and the distance between the urban center and the seismogenic fault are the same to the damage ratio, indicating they have a greater impact on the urban building damage. Specifically, greater average slope and closer distance between the urban center and the seismogenic fault leads to a higher urban building damage ratio. In addition, our analysis of the influencing factors of building damage is only based on topography and geological structure, but building damage is also related to local geological structure, climate, construction structures and materials, which needs further research [32,33].

### 4.3. SAR Image Parameters and the Identification Results

**Figure 9:**
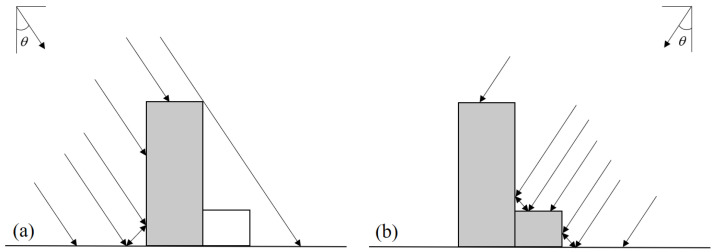
Building echo examples. Gray-filled rectangles are the buildings that receive radar microwave signal, and the white-filled rectangle is the building that does not receive radar microwave signal. Black arrows are radar signals. (**a**) The microwave signal direction is on the left of the building. (**b**) The microwave signal direction is on the right of the building.

In the selected study areas, buildings are tightly packed and mostly low-rise buildings [1]. If there are middle or high-rise buildings around, they may block the radar signals emitted to low-rise buildings, as shown in Figure 9a, resulting in failure in identification of low-rise buildings [39]. However, illuminating the radar wave in another direction can capture these sheltered low-rise buildings well. As shown in Figure 9b, in the radar line of sight direction, the middle and high-rise building is behind the low-rise building and does not form occlusion. SAR amplitude images can clearly reflect the echo signals of the two buildings. Therefore, the use of multi-geometric information to identify damaged buildings can improve identification and the accuracy of change detection.

**Figure 10:**
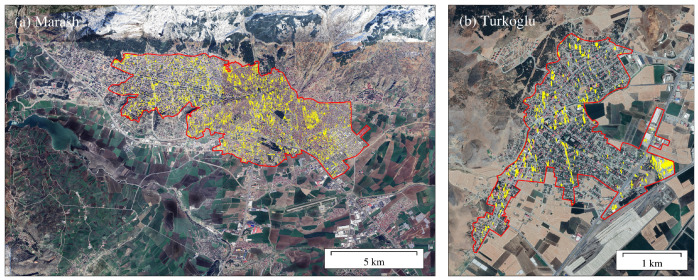
Urban building damage identification results with ascending data. The red polygon delineates the urban boundary. Yellow-filled polygons are the identified damaged buildings.

We processed two ascending images covering the two cities of Marash and Turkoglu. The image time spans from 4 February 2023 to 28 February 2023, covering the time that the earthquake happened (Figure 6, Table 1). Then we detected the damaged buildings in the urban area (Figure 10). We fused this result with the identification result of the descending images to obtain a new damage ratio and sort. As shown in Table 6, the damage ratio sort is the same as the artificial intelligence identification result of the Microsoft team, which damage ratio from high to low is Marash, Nurdagi, Turkoglu, Antakya and Islahiye. The result shows that multi-geometric fusion can improve the identification accuracy.

The intensity value of a SAR image reflects the scattering intensity of ground objects to microwave. There are two influencing factors. One is the working parameters of radar system, such as radar working wavelength, incident angle, polarization mode. The other is the parameters of ground objects, such as surface roughness and complex permittivity [40]. The resolution has a greater impact on the ground wavelength than the signal transmitting unit. The ground target of each received signal can be divided into multiple resolution units. The echo signal received by each unit is the vector superposition of all echo signals in the unit. Signals are not only from the target ground object, and the irradiation of the target ground object is mixed with the echo signal of other ground objects. These form speckle noise, which reduce the radiation resolution and spatial resolution of SAR images [40,41]. In conclusion, the radar working wavelength has little effect on the scattering intensity of ground objects, but the resolution has a great influence on the quality of SAR amplitude images.

We used the medium-resolution (14 m × 14 m) SAR data in the study. One resolution unit may contain multiple buildings, so the change of a single building may be impossible to cause the change of the pixel gray value. Therefore, the experimental results cannot achieve a recognition accuracy as high as that using high-resolution optical images or SAR images. Subsequently, we can choose higher resolution images to obtain higher accuracy recognition results. If the research period is long, selecting images with a long-time range to calculate the mean value of images pre-earthquake and post-earthquake can reduce random noise. In addition, when the image resolution is improved, the inland elements of the resolution unit are single, and the recognition results can be accurate to a single building. Currently, we can classify the recognition results into complete damage buildings and incomplete damage buildings, which are favorable for disaster rescue.

## 5. Conclusions

The 2023 earthquakes in Turkey, measuring Mw7.8 and Mw7.7, were the strongest recorded since 1939 and caused extensive damage to cities in central and southern regions. Our study utilized the change detection method to identify damage in five heavily impacted cities from Sentinel-1 images. Damage ratios were calculated based on the affected urban area and building damage area. The largest proportion of damage was seen in Nurdagi at 18.93%, while Islahiye had the smallest proportion at 7.59%. Nurdagi showed the largest average surface deformation at 0.48 m while Antakya had the smallest at 0.09 m. The study’s results were verified by comparing with Microsoft’s artificial intelligence recognition results. Furthermore, the study analyzed the factors contributing to building damage after the earthquakes. We found that, among the four factors, which are average slope, distance between urban center and seismogenic fault, distance between urban center and epicenter and surface deformation magnitude, the first two have a greater impact on the urban building damage. Additionally, this study analyzed the effects of SAR image parameters on building damage detection accuracy. The experiment of ascending orbit image shows that the multi-geometry detection results have a good supplement to the single-geometry detection results. Higher image resolution leads to higher recognition results. Despite the medium-resolution, Sentinel-1’s high spatial resolution and free distribution make it a valuable tool for assessing large-scale building damage.

## Figures and Tables

**Table 1:** Data information.

Sensor	Band (Wavelength/cm)	Spatial Perpendicular Baseline (m)	Frames Width (km)	Track Direction	Frames	Incidence Angle (°)	Azimuth Angle (°)	Image Resolution (m)	Temporal
Sentinel-1	C (5.63)	106.49	250	Descending	DT21	39.56	193.25	5 × 20 (Rg × Az)	29 January 2023 10 February 2023
	C (5.63)	106.49	250	Ascending	AT116	39.53	90.00	5 × 20 (Rg × Az)	4 February 2023 28 February 2023

**Table 2:** Data processing parameters.

**POT**	**Search Window Size (Rg × Az/Pixel)**	**Moving Step (Rg × Az/Pixel)**	**Oversampling Factor**	
	300 × 60	5 × 1	2	
**D-InSAR**	**Multi-look number of interferogram pair (Rg:Az)**	**Spatial perpendicular baseline (m)**	**Filtering window size (Rg × Az/pixel)**	**Filtering factor**
	5:1	106.42	128 × 128	0.2

**Table 3:** Building damage in the five urban areas.

City	Urban Area (km^2^)	Damaged Buildings Area (km^2^)	Damage Ratio (%)
Nurdagi	2.58	0.49	18.93
Marash	37.92	6.59	17.37
Antakya	14.96	2.14	14.28
Turkoglu	3.07	0.39	12.79
Islahiye	3.32	0.25	7.59

**Table 4:** Surface deformation of the five urban areas.

City	Mean Deformation (m) Obtained by POT	Mean Deformation (m) Obtained by D-InSAR	Mean Deformation (m)
Turkoglu	0.46	0.54	0.50
Marash	0.40	0.52	0.46
Nurdagi	0.48	0.44	0.46
Islahiye	0.26	0.31	0.28
Antakya	0.09	0.09	0.09

**Table 5:** Topographic and geological information of the five urban areas.

City	Mean Deformation (m)	Average Slope (°)	Distance from the Fault (km)	Distance from the Hypocenter (km)	Damage Ratio (%)
Nurdagi	0.46	2.3	0.6	10.86	18.93
Marash	0.46	4.9	15.2	44.85	17.37
Antakya	0.09	2.9	5.2	124.92	14.28
Turkoglu	0.50	2.4	2.9	21.82	12.79
Islahiye	0.28	3.4	1.4	25.41	7.59

**Table 6:** Building damage in the five urban areas with multi-geometric fusion.

City	Urban Area (km^2^)	Damaged Buildings Area (km^2^) (Descending)	Damaged Buildings Area (km^2^) (Ascending)	Damage Ratio (%)
Marash	37.92	6.59	5.80	23.39
Nurdagi	2.58	0.49	/	18.93
Turkoglu	3.07	0.39	0.25	14.33
Antakya	14.96	2.14	/	14.28
Islahiye	3.32	0.25	/	7.59

## Data Availability

The Sentinel-1 data used in this study are copyrighted by the European Space Agency (http://scihub.copernicus.eu/dhus, accessed on 1 March 2023). The moment tensor solution is from the Global Centroid Moment Tensor (http://www.globalcmt.org/CMTsearch.html, accessed on 1 April 2023). The GAMMA commercial software was obtained from https://www.gamma-rs.ch/software, accessed on 22 February 2023.
